# Angiotensin II as a Vasopressor for Perioperative Hypotension in Solid Organ Transplant

**DOI:** 10.3390/biomedicines12081817

**Published:** 2024-08-09

**Authors:** Scott T. Benken, Riya Thomas, Dustin R. Fraidenburg, Jamie J. Benken

**Affiliations:** 1 Department of Pharmacy Practice, University of Illinois Chicago College of Pharmacy, Chicago, IL 60612, USA; riya.christina@gmail.com (R.T.); jjosep9@uic.edu (J.J.B.); 2 Department of Medicine, Division of Pulmonary, Critical Care, Sleep, and Allergy, University of Illinois Chicago College of Medicine, Chicago, IL 60612, USA; dfraiden@uic.edu

**Keywords:** angiotensin II, Giapreza, hypotension, shock, solid organ transplant, kidney transplant, liver transplant, heart transplant, lung transplant, vasopressor

## Abstract

During the perioperative period of transplantation, patients experience hypotension secondary to the side effects of anesthesia, surgical stress, inflammatory triggering, and intraoperative fluid shifts, among others causes. Vasopressor support, in this context, must reverse systemic hypotension, but ideally, the agents used should benefit allograft function and avoid the adverse events commonly seen after transplantation. Traditional therapies to reverse hypotension include catecholamine vasopressors (norepinephrine, epinephrine, dopamine, and phenylephrine), but their utility is limited when considering allograft complications and adverse events such as arrhythmias with agents with beta-adrenergic properties. Synthetic angiotensin II (AT2S–[Giapreza]) is a novel vasopressor indicated for distributive shock with a unique mechanism of action as an angiotensin receptor agonist restoring balance to an often-disrupted renin angiotensin aldosterone system. Additionally, AT2S provides a balanced afferent and efferent arteriole vasoconstriction at the level of the kidney and could avoid the arrhythmic complications of a beta-adrenergic agonist. While the data, to date, are limited, AT2S has demonstrated safety in case reports, pilot studies, and small series in the kidney, liver, heart, and lung transplant populations. There are physiologic and hemodynamic reasons why AT2S could be a more utilized agent in these populations, but further investigation is warranted.

## 1. Introduction: Angiotensin II as a Vasopressor

**Figure 1:**
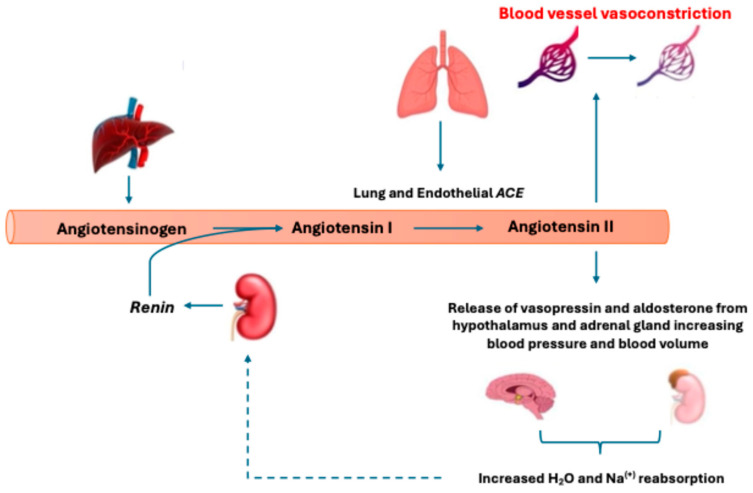
Depiction of the renin–angiotensin–aldosterone System (RAAS) and classic metabolic pathway. Low systemic blood pressure (BP) will stimulate kidney renin release to promote angiotensin I (AngI) cleavage from liver-expressed angiotensinogen. This is followed by converting AngI to angiotensin II (AngII) via angiotensin-converting enzyme (ACE). AngII then acts to increase BP via the angiotensin II type 1 receptor through direct vasoconstrictive actions and secondary signaling increasing vasopressin and aldosterone release. The production of AngII then inhibits further renin release through negative biofeedback (shown as a dotted line).

Synthetic angiotensin II (AT2S–[Giapreza]) is a novel vasopressor indicated for patients experiencing distributive shock [1]. Mechanistically, pharmacologic AT2S mimics its endogenous octapeptide counterpart primarily by activating the angiotensin II type 1 G-coupled protein receptor [2,3,4,5]. This activation leads to the calcium-dependent phosphorylation of myosin, leading to vascular smooth muscle contraction, increased systemic vascular resistance (SVR), and increased blood pressure (BP) (Figure 1) [6]. In 2017, the United States (US) Food and Drug Administration (FDA) approved AT2S after the completion of the Angiotensin II for the Treatment of High-Output Shock 3 (ATHOS-3) trial [7]. This study randomized subjects with fluid refractory, catecholamine-resistant distributive shock to receive either AT2S or placebo. The primary outcome was the incidence of mean arterial pressure (MAP) goal attainment within 3 h (defined as a MAP increase of ≥10 mmHg from baseline or an overall increase to ≥75 mmHg). In 70% of subjects receiving AT2S, the goal MAP was achieved within a median of 5 min, with sustained effects lasting throughout the study. Rapid MAP goal attainment allowed significant dose reductions to the study drug and background catecholamine vasopressor within 30 min of AT2S initiation until protocol discontinuation at 48 h. Post hoc analyses of the ATHOS-3 trial found improved 28-day survival, greater BP response, and earlier liberation from renal replacement therapy (RRT) in patients receiving AT2S who required RRT at the time of drug initiation or who had serum renin levels above the median [8,9]. While AT2S has been available since 2017, it has yet to be extensively incorporated into international vasopressor guidance, particularly in transplant populations [10,11]. Given the unique mechanism of action and potential disruptions to the renin–angiotensin–aldosterone system (RAAS) pathway in the peri-transplant patient, exploration into this population is warranted. Table 1 summarizes the published literature to date in the organ transplant population.

## 2. Abdominal Transplantation: Implications for Kidney and Liver Transplantation

### 2.1. Pathophysiologic/Hemodynamic Rationale for Use in Kidney Transplant

Hemodynamic autoregulation is impaired in the transplanted denervated kidney, making renal blood flow (RBF) dependent on systemic blood flow. Systemic blood flow is sustained by ensuring adequate cardiac output (CO) and SVR. In the perioperative setting of kidney transplant (KT), in patients with adequate CO, BP stability is, therefore, dependent on increasing SVR with vasopressors [12,13,14,15].

**Figure 2:**
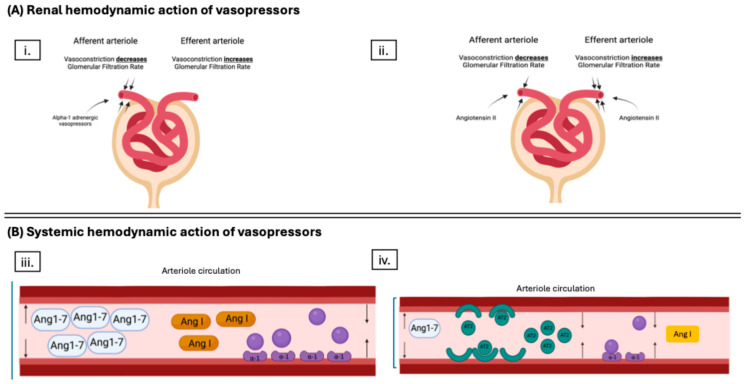
Schematic of the mechanism of action of different vasopressors during hypotension surrounding kidney transplant (KT). Panel (**A**) demonstrates the primary effect of the different vasopressors on the microcirculation of the kidney. Subpanel (i.) shows a primary afferent arteriole vasoconstriction with alpha-1 adrenergic agents. Subpanel (ii.) shows a primary efferent arteriole vasoconstriction with angiotensin II (AT2S). The difference is vital during kidney transplant as the increased endothelin, reduced nitric oxide production, and increased endothelial reactivity to vasoactive substances lead to afferent arteriole vasoconstriction, which could be worsened if using alpha-1 adrenergic vasopressors. Panel (**B**), subpanel (iii.) shows that while catecholamine vasopressors work through the alpha-1 adrenergic receptor to cause arteriole vasoconstriction, they do not restore imbalances to the renin aldosterone angiotensin system, which could lead to increased amounts of vasodilating byproduct of angiotensin I (AngI) metabolism, angiotensin-1-7 (Ang1-7), leading to ineffective action as a vasopressor. Subpanel (iv.) demonstrates both the vasoconstrictor properties of AT2S via the angiotensin II type I receptor and the restoration of AngI to Ang II balance, leading to a decrease in vasodilatory byproduct of Ang I metabolism, Ang1-7. Figure created with BioRender.com.

While no specific BP goal has been shown to be superior to another during KT, several studies have suggested achieving and maintaining a systolic blood pressure (SBP) of ≥120 mmHg with a corresponding MAP of ≥95 mmHg [13,16]. However, evidence suggests systemic BP restoration alone is insufficient to ensure adequate renal perfusion [17]. Like with other organs, kidney perfusion depends on inflow and outflow pressures (i.e., renal perfusion pressure) [18]. The inflow pressure is often assumed to be similar to systemic BP, but the post-glomerular arteriole pressure (i.e., outflow pressure) is often much lower than the systemic BP [17]. Though catecholamine vasopressors may improve systemic BP, vasoconstriction of the afferent arteriole alone with minimal constriction of the efferent arteriole may not improve but instead worsen RBF, leading to the microcirculatory dysfunctions seen in KT [18]. This is of particular concern during the reperfusion period of KT, where the activation of G-coupled protein receptors by endothelin, reduced nitric oxide production, and increased endothelial reactivity to vasoactive substances compound afferent arteriole vasoconstriction and put the new renal allograft at risk for hypoperfusion [19,20,21] (Figure 2).

Clinically, perioperative catecholamine vasopressor use in KT recipients has been associated with negative allograft outcomes, including decreased urine output after KT, slower normalization of recipient serum creatinine (SCr), an increased rate of rejection, and delayed graft function (DGF) [13,15,22,23]. It is well established that there is a significant correlation between the rates of graft failure and mortality, including short-term (e.g., 1 year) and long-term (e.g., 5-year, 10-year) endpoints, making these vasopressor outcomes of vital importance [24]. Additionally, catecholamine vasopressor exposure in KT has been associated with adverse events (AE), including an increased rate of tachycardias and insulin infusion requirements, as well as an increased length of stay and increased mortality [13,14,15,22]. These observed findings demonstrate the need for alternative therapies.

**Figure 3:**
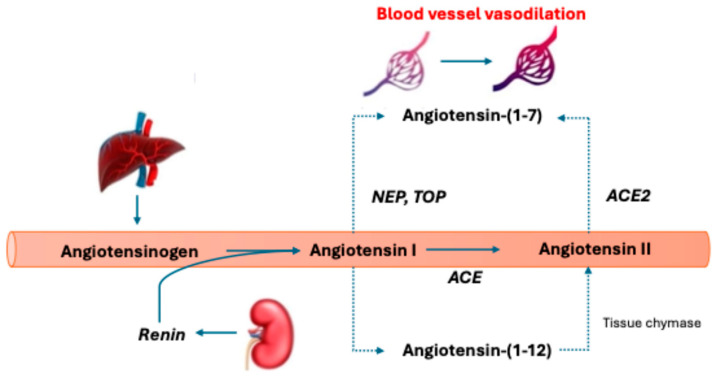
Alternative angiotensin metabolic pathway. Renin cleaves angiotensinogen to angiotensin I (AngI). This is then converted to angiotensin II (AngII) by angiotensin-converting enzyme (ACE). Angiotensin-(1-7) is created from AngI through the endopeptidases neprilysin (NEP) and thimet oligopeptidase (TOP) and AngII conversion via ACE2. Creating angiotensin-(1-7) leads to vasodilation, a decrease in fibrosis, inflammation, and increased nitric oxide levels. Angiotensin II can be created through tissue chymase via an intermediate metabolite angiotensin-(1-12). The dotted lines represent alternative metabolic pathways.

Lastly, in the KT population, brain death in the donors, cold ischemic injury, and warm reperfusion lead to the release of inflammatory cytokines that disrupt the RAAS [19,20,21]. The creation of endogenous angiotensin II (AngII) via the working capacity of the angiotensin-converting enzyme (ACE) depends on the endothelium (Figure 1) [2]. In the case of patients who experience shock, there often occurs a substantial amount of direct or indirect endothelial injury that leads to ACE deficiency and the loss of its ability to convert AngI to AngII effectively, resulting in a relative AngII insufficiency and AngI excess [8]. In states of AngI excess, there is a non-classical pathway of AngI metabolism leading to Angiotensin-(1-7) (Figure 3), which is a vasodilator that could worsen hypotensive effects in shock patients [25]. Because of the lack of termination of renin release through AngII-initiated biofeedback, there are increased levels of renin perpetuating this cycle. Interestingly, this is not a universal finding in shock, and patients can effectively be identified and categorized into high-renin and low-renin biotypes [8]. Here, it is essential to observe that, more often than not, patients who are on the KT waiting list with acute kidney injury (AKI), chronic kidney disease (CKD), and end-stage renal disease (ESRD) present with high-renin biotypes [26,27]).

Additionally, it has been found in the surgical population that several components of such surgeries can lead to a similar disruption of the RAAS. In KT patients, after brain death in the donors of the transplanted organs, there is an outpouring of inflammatory cytokines that can disrupt the endothelium, similar to the contact activation seen in cardiac surgery [28]. However, the most significant contributor to inflammation during transplantation is the ischemic injury caused by cold storage followed by warm reperfusion during renal engraftment [29]. Reperfusion of ischemic organs results in the activation of inflammatory pathways and complement cascades that increase graft immunogenicity and the numerous inflammatory mediators being expressed, which, intuitively, would lead to a disruption in AngII production secondary to a relative ACE dysfunction through the additional disruption of the endothelium [19,20,30]. There are not enough studies to quantify this relative endothelial dysfunction and its effects on ACE levels in postoperative KT patients.

Theoretically, the use of AT2S in KT could be advantageous for three significant reasons: (1) balanced renal vasoconstriction, (2) avoidance of AE, and (3) correction of RAAS derangements [2,7,31].

### 2.2. Available Angiotensin II Data in Kidney Transplant

Studies show that AT2S provides more balanced vasoconstriction within the renal vasculature and yields a net result of increased filtration fraction, which, in theory, could preserve renal function [7,9]. By avoiding preferential afferent arterial vasoconstriction and restoring the AngI:AngII balance, supplementation with synthetic AT2S may lessen the risk of reperfusion injury during KT and potentially avoid ischemia in the new allograft. This would be anticipated to be seen with better rates of allograft function after KT.

No available data in the KT space have directly observed improvements in allograft function with AT2S. In a pilot study of AT2S in 20 patients undergoing KT who experienced perioperative hypotension, DGF was observed in two of the 20 cases (10%) [12]. This was a surprising finding, as the study population was primarily deceased donor kidney transplant (DDKT) patients with a reported mean (standard deviation [SD]) cold ischemia time (CIT) of 14.7 (8.6) h. With this CIT, according to a predictive model by Irish et al., the DGF rate would have been estimated to be ~12 of 20 cases (60%), as it has been observed that there is an increased risk of DGF of 4% for every 1 h increase in CIT [32]. This risk may have been mitigated by using AT2S instead of catecholamine vasopressors. While intriguing, this study’s lack of a comparative group makes this only speculative. Additionally, unpublished retrospective data from our institution observed clinical differences in the rates of immediate graft function (IGF) (70% vs. 61.4%; *p* = 0.054) in a cohort studying comparing 20 AT2S patients to 60 previous standards of care (i.e., dopamine, phenylephrine, epinephrine) patients with perioperative hypotension surrounding KT. These findings were despite disadvantageous baseline characteristics in the AT2S group regarding CIT (14.7 ± 8.6 h vs. 7.9 ± 5.6 h; *p* < 0.001) and donor terminal serum creatinine (Scr) (1.79 ± 1.7 vs. 1.11 ± 0.86; *p* = 0.027) [33]. Lastly, another retrospective study comparing 56 AT2S patients to 49 phenylephrine patients with perioperative hypotension surrounding KT observed a benefit regarding DGF, but only in patients with prolonged CIT (i.e., greater than 14 h) (22.7% vs. 58.3%; *p* = 0.045) [34]. In that high-risk group, it was observed that patients receiving phenylephrine were at an 8.7-fold increased risk of DGF (95% 1.18 to 62.5; *p* = 0.033) compared to those who received AT2S. Perhaps those with the highest risk of allograft dysfunction should preferentially receive AT2S.

Many of the AEs seen with vasopressors are thought to be secondary to beta1-adrenergic stimulation. Other catecholamine vasopressors with beta1-adrenergic stimulation (e.g., norepinephrine [NE], dopamine [DA], epinephrine [EPI]) increase the rate of spontaneous depolarization in cardiac fibers via the cyclic adenosine monophosphate system that may lead to tachyarrhythmias [35]. Studies show that tachyarrhythmias, specifically atrial fibrillation (AF), are prevalent in the KT population [36,37]. It has been observed that up to 7% of renal allograft recipients will develop AF within 3 years of transplant, with the highest risk being the perioperative period of transplant [37]. Further, it was observed that developing AF following KT was independently associated with increased mortality and death-censored graft failure. This association between catecholamine vasopressors, the development of arrhythmias, and their negative correlation with outcomes is consistent with data in non-KT populations as well [38,39,40]. In our internal data, we observed a superior safety profile for AT2S compared to catecholamine vasopressors (perioperative arrhythmias 5% vs. 28.3%; *p* = 0.03 and digital ischemia 0% vs. 18.3%; *p* = 0.031) [33]. These observations were consistent after propensity-matched weighting to account for allograft risk stratification [33]. While these data are encouraging, the study population was relatively small, which could have led to imprecision in the statistical observations.

### 2.3. Pathophysiologic/Hemodynamic Rationale for Use in Liver Transplant

During liver transplantation (LT), recipients often experience low SVR with normal or high CO with an increase in the frequency of these hemodynamic alterations with the severity of pre-transplant liver disease and/or pre-transplant end-stage renal disease (ESRD) requiring RRT [41,42,43]. High doses of catecholamines such as NE and EPI are typically infused to maintain mean arterial pressure (MAP) and organ perfusion during LT [10,41]. If these initial therapies fail, vasopressin can be considered as a second-line therapy to decrease the amount of NE or EPI [10]. There is no comment on using phenylephrine (PE) by international guidance [10]. While these recommendations stem from international guidance, they are extrapolated from non-liver transplant data, primarily those of the septic shock population. As noted above in KT, similar concerns would exist in this population, including microvascular perfusion issues in the kidneys and other organs [9,13,14,15,22,23,44] and arrhythmias [14,45,46,47]. Vasopressin has yet to demonstrate significant, sustained impacts on renal outcomes in patients with shock [48,49,50]. It would be unexpected for vasopressin to have different outcomes in this population, leaving limited alternative options. Non-catecholamine rescue therapies have, thus far, in LT, been limited to the off-label use of methylene blue or hydroxocobalamin, and given the dearth of information surrounding their use, these agents are not widely utilized [51,52,53]. New agents are needed, particularly those that can reduce the rate of renal complications post-LT.

Acute kidney injury (AKI) after LT is frequent, occurring in 50–80% of patients after LT [54]. Additionally, the development of AKI post-LT significantly reduces patient and graft survival [54,55,56,57]. Given the implication of catecholamine vasopressors in the development of AKI, using a different agent with an alternative mechanism upfront may reduce postoperative AKI after LT. Additionally, the RAAS is known to be altered in cirrhosis, starting with increased angiotensinogen expression, which triggers alternative pathways that contribute to the pathophysiology of end-stage liver disease (ESLD). A known upregulation of the RAAS contributes to the progression of hepatic fibrosis, portal hypertension, and sodium and water derangements [58,59]. Additionally, there is a heightened counter-regulatory alternative metabolic pathway that is triggered during ESLD, which leads to the metabolism of physiologic AngII to a vasodilatory byproduct, Ang-(1-7) (Figure 3), leaving a patient with ESLD in a chronically vasodilated/hypotensive state [58,60]. These levels of vasodilatory byproducts return to normal once the circulatory abnormality associated with advanced cirrhosis has corrected, making time to hemodynamic correction a vital variable in the perioperative period of LT [58,61]. In the ATHOS-3 Phase 3 clinical trial, AT2S was shown to more frequently lead to MAP goal attainment than the standard of care (i.e., NE), with a median duration of MAP goal attainment of 5 min for patients on AT2S, making this agent appealing for the LT population [7].

### 2.4. Available Angiotensin II Data in Liver Transplant

Current data reporting the use of AT2S in LT are limited to case reports [62,63]. In the first case report by Wieruszewski et al., AT2S was used to manage a vasoplegic shock syndrome in a 34-year-old female with a post-orthotopic heart transplant and combined deceased donor liver transplant (DDLT) status [63]. Two weeks after surgery, the patient faced significant hemodynamic challenges despite high doses of NE, vasopressin, and oral midodrine. The patient was determined to have left ventricular outflow tract obstruction with systolic anterior motion (SAM) of the mitral leaflet and severe tricuspid regurgitation requiring repair. The treatment team wanted to avoid beta1-agonism, given the patient’s complex cardiac physiology. AT2S was started at 20 ng/kg/min, restoring systemic pressures within minutes, allowing the patient to go to the operating room for a successful myectomy and tricuspid valve repair. In the second case by Patel et al., AT2S was used as a salvage therapy for a patient on three vasopressors (i.e., NE, vasopressin, EPI) with an intraabdominal abscess proceeding LT. AT2S was started at 10 ng/kg/min, allowing for a down-titration of the background vasopressors and adequate hemodynamic stability to position the team to proceed with the LT. The patient was successfully transplanted, weaned from vasopressors within 32 h, and discharged successfully from the hospital within 2 weeks. Currently, a randomized controlled study is underway evaluating the effectiveness of AT2S as a second-line intraoperative vasopressor during DDLT [54]. The results are pending.

## 3. Thoracic Transplantation: Implications for Heart and Lung Transplantation

### 3.1. Pathophysiologic/Hemodynamic Rationale for Use in Heart Transplant

As far back as the 1960s, there is a report of the successful use of AT2S in the treatment of shock during cardiac surgery [64]. Further research in the 1990s clearly showed that angiotensin plays an important role in cardiac transplantation [65]. It was found that renin substrate, as a precursor of AngII, was significantly increased on day 1 of cardiac transplant and remained elevated at the 12-week follow-up, which was thought to be important to the early, adaptive proliferative changes in these recipients. Case reports around this time also supported the use of AT2S in cardiac surgery [66]. While these reports proposed a distinct role of AT2S treatment in cardiac surgery, the use of AT2S appears to have been hindered by limited high-quality evidence and poor availability until the late 2010s.

AT2S may have a particular benefit in vasoplegic shock following cardiopulmonary bypass (CPB), which occurs in about a quarter of subjects undergoing major cardiac surgery [67]. In these cases, blood bypasses the pulmonary circulation where, normally, AngI would be exposed to ACE and converted to AngII, creating a relative AngII deficiency [68]. Endopeptidases can convert excess AngI to angiotensin 1-7, resulting in vasodilation and increasing hypotension. This imbalance hypothesis is further strengthened when recognizing that prolonged duration of CPB increases the risk of vasoplegic shock [69]. ACE inhibitor use prior to major cardiac surgery has also been identified as an independent predictor of post-CPB vasoplegic shock [70].

### 3.2. Available Angiotensin II Data in Heart Transplant

Supplementing with synthetic AT2S presents an appealing option as an adjunct to catecholamine vasopressors. Indeed, in the landmark trial leading to approval of synthetic AT2S, 16 subjects included in the study had shock as the result of vasoplegic syndrome post-cardiothoracic surgery [7]. The post-hoc analysis of this study showed a particular benefit in subjects with vasoplegia following cardiac surgery treated with AT2S as compared to placebo [71]. In these cases, 89% of subjects achieved a MAP of 75 mm Hg or higher or had an increase of at least 10 mm Hg from baseline at 3 without adjusting the catecholamine doses. Since FDA approval, subsequent case reports have shown a benefit of AT2S in vasoplegic shock at doses up to the maximum of 80 ng/kg, mainly when the hypotension is refractory, requiring multiple vasoactive agents [63,72,73,74]. These cases suggest that AT2S can be used as an adjunctive agent to reduce the overall catecholamine requirement and reduce shock days, yet randomized trials are lacking. A case report also suggests benefits in patients with vasoplegia as a result of LVAD implantation [75]. Though thrombotic complications remain a particular concern, this patient’s hypotension rapidly improved, and the treatment of heart failure resulted in discharge from the hospital with a future successful heart transplant noted. A post-marketing study assessing the safety and efficacy of AT2S in shock included 28 subjects with postoperative vasoplegia [76]. Subjects with postoperative vasoplegia showed a similar response to AT2S as the overall population, with nearly 2/3 of these subjects reaching a MAP of 65 or greater within 3 h of AT2S initiation. While this subgroup was not reported individually, the overall outcomes showed that AT2S responders had improved 30-day survival.

The mounting evidence demonstrated in case reports and clinical trial subgroups has provided the necessary energy to study this robustly. A study examining the implementation of a vasoplegia protocol utilizing AT2S found a significant reduction in requirements for norepinephrine and dobutamine [77]. Despite increased MAP with early implementation of AT2S, these vasoplegic shock patients continue to have a high mortality. The results of a feasibility trial have also been reported, comparing AT2S to norepinephrine infusion as the initial agent used intraoperatively and for up to 48 h postoperatively to achieve a MAP of 70–80 mmHg [78]. AT2S achieved the goal MAP at a similar rate to norepinephrine without any increase in significant adverse outcomes. There was a trend toward reduced AKI in the first 7 days postoperatively in the AT2S group, compared to the norepinephrine group, with a reduced hospital length of stay. These results open the door for future studies examining the use of AT2S in cardiac surgery and heart transplantation to improve clinical outcomes and reduce critical adverse events such as AKI.

Little is known about how AT2S may impact graft function and longevity in heart transplants. It is known that AT2S is involved in cardiac remodeling and, in small rodent models, prevents atrophy of transplanted heart tissue [79,80]. AT2S is intricately involved in many cardiac cell processes and, therefore, could be implicated in both the early and late-stage complications of cardiac transplantation [81]. As our understanding of the role of AT2S infusion in cardiac surgery and heart transplant evolves, AT2S will likely continue to have an increased role in the early treatment of refractory shock and, potentially, as a preferred agent to support cardiac function and prevent AKI after high-risk cardiac surgeries.

### 3.3. Pathophysiologic/Hemodynamic Rationale for Use in Lung Transplant

Similar to a heart transplant, a bilateral lung transplant requires cardiopulmonary bypass for successful explant and transplantation, which carries the risk of a significant imbalance of AngI, angiotensin 1-7, and AngII. This puts patients at a similar risk of vasoplegic shock, yet a single lung transplant can avoid cardiopulmonary bypass and, therefore, lessen this risk. Long-term outcomes tend to favor double lung transplantation, which has remained the preferred approach, outnumbering single lung transplantation 4 to 1 annually [82,83]. There is a concern, however, that the renin–angiotensin system has been linked to pulmonary fibrosis, with AngII being a primary culprit [84,85]. AngII also appears to be highly active in chronic lung allograft dysfunction, a fibrotic type of chronic rejection that is the primary cause of death in lung transplant recipients [86]. Elevated levels of AngII have also been described acutely during lung transplantation immediately following CPB and returning to baseline levels after 6 h [87]. The early elevation of AngII was thought to stimulate pro-inflammatory and pro-fibrotic signaling in the lung. These effects of AngII in the lung caution the use of pharmacologic AT2S treatment in lung transplant recipients, yet little is known about this therapy’s direct, short-term effects, particularly when treating early complications such as vasoplegic shock.

### 3.4. Available Angiotensin II Data in Lung Transplant

In one case study, a patient with a bilateral lung transplant developed vasodilatory shock after receiving anti-thymocyte globulin requiring rapidly escalating catecholamines [74]. In this case, AT2S resulted in a swift and significant blood pressure response. AT2S was started at a reduced dose (10 ng/kg) to prevent rapid increases in pulmonary vascular resistance and worsening pulmonary hypertension. However, this complication was not encountered, as central venous pressure (CVP) remained stable throughout the treatment. While there appears to be a role for AT2S in lung transplant recipients experiencing vasoplegic shock after coming off CPB, the impacts of this therapy on graft perfusion, acute inflammation, fibrosis, and chronic rejection remains unknown.

**Table 1:** Observed outcomes of AT2S in solid organ transplant.

Organ	Outcomes	Citation
Kidney	A pilot study of 20 KT patients receiving AT2S first line as a vasopressor. Median duration of AT2S usage was 1 h intraoperatively and 26.5 h postoperatively. Only one patient needed additional vasopressor support. No adverse events were reported.	[12]
	A propensity-matched study of 20 patients receiving AT2S compared to 20 patients receiving catecholamine vasopressors. Similar duration of intraoperative vasopressor usage, but longer median duration of total vasopressor usage, was found with AT2S (47.5 h versus 13.8 h; *p* = 0.05). Lower rates of arrhythmias with AT2S (5%) compared to catecholamine vasopressors (30%; *p* = 0.04). Similar allograft outcomes.	[33]
	A retrospective study comparing 56 AT2S patients to 49 phenylephrine (PE) patients with perioperative hypotension surrounding KT. Median (IQR) duration of vasopressor use was 12.8 (2.82–52.0) h in the AT2S group compared to 13.7 (2.72–32.8) h in the phenylephrine group (*p* = 0.646). There was a decreased need for additional vasopressors when using AT2S as the primary vasopressor compared to PE (2 [3.6%] vs. 18 [36.7%]; *p* < 0.001). There was an observed benefit regarding rates of DGF in patients with prolonged CIT (greater than 14 h) receiving AT2S (22.7%) versus PE (58.3%; *p* = 0.045).	[34]
Liver	A case report of a 34-year-old female with vasoplegic shock syndrome after an orthotopic heart transplant and combined deceased donor LT. AT2S initiated at 20 ng/kg/min, restoring systemic pressures within minutes, and allowing successful myectomy and tricuspid valve repair.	[63]
	A case report of a patient on three vasopressors due to intraabdominal abscess preceding LT. AT2S started at 10 ng/kg/min for refractory shock, which allowed for down-titration of background vasopressors, providing hemodynamic stability for LT. The result led to successful LT, weaning off vasopressors within 32 h, and discharge from the hospital within 2 weeks.	[53]
	A randomized controlled study is underway evaluating AT2S as a second-line intraoperative vasopressor during deceased donor LT. The study drug infusion is initiated on reaching a norepinephrine dose of 0.05 µg/kg/min and titrated per protocol. The primary outcome is the dose of norepinephrine required to maintain a mean arterial pressure ≥65 mm Hg. Secondary outcomes include vasopressin or epinephrine requirement and duration of hypotension. Safety outcomes include incidence of thromboembolism within 48 h of the end of surgery and severe hypertension.	[54]
Heart	A trial of 16 subjects in the ATHOS-3 study treated with AT2S or standard of care for vasoplegic syndrome post-cardiothoracic surgery. Mean arterial pressure response was achieved in 8 (88.9%) patients in the angiotensin II group compared with 0 (0%) patients in the placebo group (*p* = 0.0021). At 12 h, the median background vasopressor dose had decreased from baseline by 76.5% in the AT2S group compared with an increase of 7.8% in the standard of care group (*p* = 0.0013). No venous or arterial thrombotic events were reported.	[71]
	Post-marketing study, of which 28 subjects had postoperative vasoplegia treated with AT2S. Nearly 2/3 reached a MAP of 65 or greater within 3 h. Improved 30-day survival was observed in patients who responded to AT2S infusions.	[76]
	A feasibility study of 60 subjects comparing AT2S to NE infusion in cardiac surgery and heart transplant patients reported achieving similar MAP without increased adverse outcomes. Study drug duration was median (IQR (range)) 217 min (160–270) vs. 185 min (135–301 (0–480)) (*p* = 0.78) intraoperatively, and 5 h (0–16 (0–48)) vs. 14.5 h (4.8–29 (0–48)) (*p* = 0.075) postoperatively for AT2S and NE, respectively. The mean arterial pressure target was achieved postoperatively in 25 of 28 (89%) of the AT2S group and 27 of 32 (84%) of the NE group. There was a trend toward reduced AKI and shorter hospital stay in AT2S group.	[78]
Lung	A case series including one patient receiving a bilateral lung transplant. Following anti-thymocyte globulin administration, a patient developed vasodilatory shock, necessitating rapid catecholamine escalation despite multiple vasopressors and adjunct agents. AT2S was cautiously initiated at 10 ng/kg/min to prevent worsening pulmonary hypertension. Despite concerns, central venous pressure remained stable. AT2S promptly restored blood pressure, allowing for down-titration of background vasopressors, suggesting potential efficacy in managing post-CPB vasoplegic shock in lung transplant recipients.	[74]

AKI = acute kidney injury, AT2S = pharmacologic angiotensin II, CIT = cold ischemic time, CPB = cardiopulmonary bypass, DGF = delayed graft function, IQR = interquartile range, KT = kidney transplant, LT = liver transplant, MAP = mean arterial pressure, NE = norepinephrine, PE = phenylephrine.

## 4. Additional Considerations and Future Directions

### 4.1. Cost Considerations

As detailed above, there are pathophysiologic and hemodynamic rationales for the use of AT2S in solid organ transplant (SOT) along with a growing dataset on its use in transplant patients. Along with these rationales and data, there are essential costs to consider. To administer vasopressors at a dose equivalent to 0.05 mcg/kg/min of norepinephrine in a 70-kilogram (kg) patient for 60 min, the costs for common vasopressor options are listed in Table 2. Compared to other continuous vasopressor options, AT2S, terlipressin, and vasopressin cost more than NE, EPI, DA, and PE. An analysis assessed the cost-effectiveness of adding AT2S to the standard of care (SOC) for severe distributive shock in the US critical care setting from a US payer perspective [68]. The results of this analysis showed that the addition of AT2S saved 0.08 lives at day 28 compared to SOC alone, the cost per life saved was estimated to be USD 108,884, and the addition of AT2S to SOC was projected to result in a gain of 0.96 life years and 0.66 quality-adjusted life years (QALYs). This resulted in an incremental cost-effectiveness ratio of USD 12,843 per QALY. The probability of AT2S being cost-effective at a threshold of USD 50,000 per QALY was 86%. The authors concluded that AT2S is cost-effective at acceptable thresholds to treat severe distributive shock.

While a cost-effectiveness analysis of AT2S in SOT has not been performed, using AT2S may reduce certain costly complications that occur post-transplant.

As previously reviewed, AT2S, when used for perioperative hypotension surrounding KT, may be beneficial in preventing DGF, especially in those at the highest risk for DGF. Investigating the cost impact of DGF, a retrospective analysis was performed of hospital healthcare resource utilization and cost in KT patients [88]. This study included 12,097 KT admissions between 2014 and 2018 across 56 hospitals, including low-volume and high-volume centers and urban and rural settings. Approximately one quarter of these KT recipients experienced DGF. Patients who experienced DGF had a longer mean ± SD initial length of stay (LOS) (12.1 ± 12.9 vs. 7.4 ± 3.8 days, *p* < 0.0001), more frequent intensive care unit (ICU) admissions (59% vs. 56%, *p* = 0.025), longer ICU stays (4 ± 11.2 vs. 2 ± 3.2 days, *p* < 0.0001), and a higher 90-day readmission rate (46% vs. 30%, *p* < 0.001). Patients with DGF had significantly higher costs for the initial hospitalization and readmissions over the following 90 days ($130,492 ± 56,701 vs. $112,598 ± 57,675, *p* < 0.001). The results demonstrated that DGF was associated with an approximate USD 18,000 increase in mean cost, 6 additional days of hospitalization, and 2 additional days of ICU stay. On average, hospitals saw a greater than 10% increase in costs with DGF.

As previously discussed, AF is a concerning adverse event observed in transplant patients when beta-1 adrenergic vasopressors are utilized. The use of AT2S may avoid this complication. The cost of postoperative atrial fibrillation (POAF) following transplant is not well-reported; however, POAF cost has been studied in other settings. A retrospective propensity-matched, multivariate regression analysis was performed to compare 1-year outcomes and costs in 2096 patients with (n = 549) and without (n = 1547) POAF following coronary artery bypass graft surgery (CABG) [89]. For the index CABG hospitalization, patients who developed POAF had a longer postoperative length of stay (+3.9 days) and higher hospitalization costs (+$13,993) than patients who did not develop POAF. At 1 year, POAF patients had more than twice the adjusted odds of dying (*p* < 0.01).

Although AT2S may have benefits in reducing certain costly postoperative complications, it is more expensive than other vasopressor options. Therefore, specific measures may be necessary to restrict its use and minimize medication waste. The short stability can lead to delays in therapy while waiting for the medication to be made upon ordering and increased medication waste as admixtures reach expiration. The current package labeling provides a 24 h expiration date after intravenous admixture for AT2S infusions. This type of issue is not unique to AT2S. A study evaluated the extended stability of vasopressin at 90 days and the impact of extended stability on time to administration and medication waste at an academic medical center [90]. The study results showed that vasopressin was stable at 90 days. Based on this stability, vasopressin preparation was moved to batching. The cost savings were determined by a wastage study in the month prior to the implementation of vasopressin batching. In that month, 24 vasopressin bags were discarded, resulting in an estimated annual cost saving of approximately USD 185,300 if 100% of the expired product could be used with new extended stability data. Having the medication ready via batching, the time from pharmacist order verification to nursing administration scan decreased significantly from 26 ± 13 min to 4 ± 6 min (*p* < 0.01). Current labeling states that admixtures of AT2S should be discarded 24 h after preparation. However, a stability study of AT2S showed that it is stable under refrigeration for up to 5 days [91]. These extended stability data may diminish the amount of wasted AT2S and allow for a reduced time to administration. Alternative options would be for AT2S to be prepared in the operation room by the anesthesia team. The extended stability data for AT2S should allow for reduced medication waste and, thereby, cost savings.

### 4.2. Future Directions

Studies have shown the benefits of using AT2S in high-renin biotypes both after cardiac surgery and in the setting of septic shock [92,93]. As reviewed above in Section 2.1, patients waiting for KT will likely be high-renin biotypes. However, it may be helpful, especially if creating use criteria, to send a renin level while the patient is on the waitlist to identify those who would most benefit from AT2S if it were needed for perioperative hypotension. Additionally, as disruptions in the RAAS cascade can be seen across the various types of SOT, RASS profiling in real time may be helpful to more precisely determine the vasopressor agent selection, and if RASS dysfunction is detected, using AT2S preferentially may be warranted. While there is pathophysiologic rationale and growing data for AT2S in SOT, further studies are needed. These would include explorations into each type of SOT using AT2S as a first-line vasopressor agent and investigations with more powerful study designs such as multicenter randomized controlled studies.

## 5. Conclusions

While data, to date, are limited, AT2S has demonstrated safety in case reports, pilot studies, and small series in the kidney, liver, heart, and lung transplant populations. There are physiologic and hemodynamic reasons why AT2S could be a more utilized agent in these populations, but further investigation is warranted.

## Figures and Tables

**Table 2:** Calculated cost comparison for vasopressor agents.

Vasopressor	Cost *
Norepinephrine	USD 0.21
Epinephrine	USD 3.39
Dopamine	USD 0.37
Phenylephrine	USD 0.49
Vasopressin	USD 115.92
Angiotensin II	USD 90.72
Terlipressin	USD 27.90

* AWP = average wholesale price calculated for administering norepinephrine equivalent on 0.05 mcg/kg/min in a 70 kg patient for 60 min.
